# Identification of canine osteoarthritis using an owner‐reported questionnaire and treatment monitoring using functional mobility tests

**DOI:** 10.1111/jsap.13500

**Published:** 2022-04-06

**Authors:** A. Wright, D. M. Amodie, N. Cernicchiaro, B. D. X. Lascelles, A. M. Pavlock, C. Roberts, D. J. Bartram

**Affiliations:** ^1^ Outcomes Research, Zoetis Inc. Parsippany New Jersey 07054 USA; ^2^ Center for Outcomes Research and Epidemiology, College of Veterinary Medicine Kansas State University Manhattan Kansas 66506 USA; ^3^ Comparative Pain Research and Education Centre & Translational Research in Pain Program, College of Veterinary Medicine North Carolina State University Raleigh North Carolina 27606 USA; ^4^ AMP Research Solutions Parker Ford Pennsylvania 19457 USA; ^5^ vHive, School of Veterinary Medicine University of Surrey Guildford GU2 7AL UK

## Abstract

**Objectives:**

To investigate the diagnostic value of an owner‐completed canine osteoarthritis screening checklist to help identify previously undiagnosed osteoarthritis cases, and assess their response to carprofen treatment by monitoring pain and functional mobility.

**Materials and Methods:**

Dogs (n=500) whose owners reported ≥1 positive response to the osteoarthritis checklist were examined to identify dogs with previously undiagnosed osteoarthritis. Eligible dogs (n=133) were evaluated for pain and video mobility analysis by Helsinki Chronic Pain Index and visual analogue scale scores, respectively, following carprofen treatment, administered for 30 days (n=95) or up to 120 days (n=38). Dogs were filmed at clinics performing activities (walking, jogging, sitting/lying, walking up and down stairs), and scored at days 0, 30 and 120 using visual analogue scale by an independent blinded expert.

**Results:**

A diagnosis of osteoarthritis was confirmed by a veterinarian in 38% (188 of 500) of dogs. Balance of sensitivity and specificity across the original group of nine screening questions was optimised to approximately 88 and 71%, respectively, after elimination of three questions. Pain measured by Helsinki Chronic Pain Index and functional mobility improved over time in response to treatment with carprofen. Mean ability scores for activities significantly improved between days 0 and 30 for walking, jogging, sitting/lying and walking down stairs, and days 0 and 120 for sitting/lying and walking up stairs.

**Clinical Significance:**

More osteoarthritis cases were identified in study dogs than previous prevalence estimates, indicating the screening checklist's potential to help identify for further evaluation cases that could otherwise remain undiagnosed. Improvements in function were demonstrated after carprofen treatment.

## INTRODUCTION

Canine osteoarthritis (OA) is a degenerative joint disease (DJD) that ultimately leads to functional decline of the joint, causing lameness and chronic pain (Anderson *et al*. 2020). Prevalence of OA is higher in ageing dogs; however, it is reported around 20% of dogs over 1‐year age have OA (Johnson *et al*. 1994, Johnston 1997, Autefage & Gossellin 2007). Dog owners often overlook early signs of OA which results in underdiagnosis, a major barrier to treatment intervention (Belshaw *et al*. 2020). Owners may not associate early physical and behavioural indicators with OA pain, such as lagging on walks, reduced enthusiasm and reluctance in jumping/climbing stairs (Epstein *et al*. 2015, Lascelles *et al*. 2019). However, owner perception of subtle changes in their dog's discomfort, including changes in gait, mobility, energy and behaviour is a key component to early OA detection (Epstein *et al*. 2015, Belshaw *et al*. 2020). Several validated methods using clinical metrology instruments (CMIs) generate an OA score for dogs, but only in clinically affected animals (Brown 2007, Hercock *et al*. 2009). An owner‐completed screening checklist has been developed to identify cats likely to have DJD‐associated pain (Enomoto *et al*. 2020), but no early diagnostic methods have been developed for dogs, and OA often goes undetected in dogs that appear asymptomatic (Lee *et al*. 2020). Limited questioning methods are available for owners to help guide identification of early OA signs and improve diagnosis (Lee *et al*. 2020).

In humans, pain associated with OA in lower extremities is reported to be the leading cause of mobility impairment in older adults (Murray *et al*. 2012, Vos *et al*. 2013). In dogs, pain associated with OA is often masked as early signs are subtle, making accurate pain assessment challenging (Bell *et al*. 2014). Various CMIs (questionnaires) which rate disease outcomes, such as pain and lameness have been evaluated in studies and clinical trials (Vasseur *et al*. 1995, Innes *et al*. 2003, Hercock *et al*. 2009). Assessment of pain and function should routinely be included in canine wellness examinations; however, time is limited, and pain scoring questionnaires are rarely provided to owners as they are time‐consuming (Epstein *et al*. 2015). Canine Brief Pain Inventory, a validated, owner‐completed 11‐item questionnaire, and the statistically validated Helsinki Chronic Pain Index (HCPI), Liverpool Osteoarthritis in Dogs and Canine Orthopaedic Index which are also validated, are exceptions (Brown *et al*. 2008, Hielm‐Björkman *et al*. 2009, Walton *et al*. 2013).

Pain scales used in both human and veterinary medicine often include an assessment of function. Real‐time physical functioning assessments in veterinary medicine would be a relevant measure of pain alleviation, but they do not appear to be currently used. In considering such functional assessments, it must be realised that all activities that dogs perform daily may not improve equally and/or continue to improve over time (Lascelles *et al*. 2019). More demanding tasks such as going up and down stairs and rising from sitting/lying may take longer to improve in comparison to walking or jogging in a straight line. A visual analogue scale (VAS) is a simple and easy measurement tool that has been used to measure ability in various diseases (Hammarén *et al*. 2005, NC3Rs 2020, The Kennel Club 2020).

Optimal management of OA is dependent on early treatment intervention to prevent long‐term decline in quality of life (Rychel 2010). Case management often involves a trial period of pain relief treatment which requires monitoring of changes in clinical signs of lameness over time (Belshaw *et al*. 2020). Non‐steroidal anti‐inflammatory drugs (NSAIDs) are the most commonly prescribed treatments for OA due to the availability, ease of administration, demonstrated efficacy, pain alleviation and known safety profiles of the medication (Pelletier *et al*. 1999, Pelletier *et al*. 2000, Johnston *et al*. 2008, Innes *et al*. 2010, Holloway *et al*. 2012, Monteiro‐Steagall *et al*. 2013, Bell *et al*. 2014). While NSAIDs can be associated with some adverse events including gastrointestinal disturbances, liver and/or kidney problems, the reactions observed in most of the studies have been infrequent (McAlindon *et al*. 1993, CDC 2001). In addition, there is evidence from recent literature consisting of both blinded, placebo‐controlled studies and open‐label studies that long‐term (longer than 1 month) administration of NSAIDs provides an additive or cumulative effect on pain reduction and improvement of clinical signs over time (McAlindon *et al*. 1993, Johnston *et al*. 2008, Holloway *et al*. 2012). Alternative treatment options include anti‐nerve growth factor monoclonal antibodies which have been authorised recently in several countries for the alleviation of pain associated with OA in dogs (Enomoto *et al*. 2019).

Early detection of OA is essential to manage clinical progression, and owners need a precise, simple questioning tool to identify cases of OA early, and accurately, during routine examination. However, the clinical question of additional treatment benefit or how long to treat with NSAIDs and how to monitor in daily veterinary practise is not as well defined.

The primary objective of this study was to investigate the diagnostic value of the owner‐completed OA screening checklist tool to identify previously undiagnosed OA cases. Secondary objectives were to determine (1) whether defined functional tests of activities of daily living showed improvement with NSAID treatment and (2) whether longer duration of NSAID administration provided additional benefit, as measured by masked scored videos of activities. This was accomplished by monitoring pain and mobility through ease of performing defined functional tests of activities of daily living in dogs with previously undiagnosed OA, in response to treatment intervention with carprofen.

## MATERIALS AND METHODS

### Study design

The study design was an open‐label, blinded assessment of clinically relevant outcome measures collected in a field setting. Ten veterinary practises in the United States, ranging from the East North Central division of the Midwest (IN, IL, OH, MI) and South Atlantic division of the South (VA, NC) were recruited for participation. Study enrolment proceeded from autumn 2014 to spring 2015. All dog owners signed informed consent forms before participation in the study and the participating veterinarians adhered to best‐practise standards for providing care, specifically associated with the treatment, monitoring and management of OA in dogs.

### Study population

Dogs that were presented to the veterinary practises for routine preventive care or evaluation of lameness/stiffness and were over 1 year of age, that were not receiving any treatments (prescription or over‐the‐counter including nutraceutical, special diets, pain medications and supplement‐type products) for OA, were eligible. Dogs already diagnosed with OA and receiving NSAID therapy, corticosteroid therapy or with serious endocrine disorders were excluded.

### OA screening checklist: recruitment

A nine‐binary response (yes/no) question owner‐completed OA checklist (Table 1) was developed as a discussion tool between veterinarians and pet owners, based on real‐world clinical experience. This set of standardised questions was designed to identify behaviours potentially correlated to OA diagnosis. The OA checklist aimed to evaluate the owner's perception of OA‐associated pain in their dog. A checklist was completed by each owner in the clinic before the consultation to identify whether dogs had clinical signs of OA. Dogs whose owners answered “yes” to one or more questions were then examined by a veterinarian. Presence or absence of OA was confirmed based on clinical history and orthopaedic examination. Radiography was performed to inform diagnosis as required at the discretion of the examining veterinarian. If the OA was deemed severe enough to warrant treatment, the owner was given the choice to participate in the study if they were able to come back for follow‐up.

**Table 1:** Osteoarthritis screening checklist

Please complete the following questionnaire. Answer all questions	Yes/no
1. Does your dog limp or appear stiff after exercise?	
2. Do you think you dog shows signs of pain?	
3. Is your dog reluctant to climb stairs or jump?	
4. Does your dog have difficulty in rising from a resting position?	
5. Have you noticed a change in your dog's behaviour?	
6. Does your dog tyre easily or lag behind during walks?	
7. Has your dog ever been injured?	
8. Have you ever given your dog medication for pain?	
9. Has your dog gained weight in the last year?	

### Functional tests

Each veterinary clinic was asked to identify up to 20 dogs that met these criteria during their daily practise. The total enrolment goal was 20 dogs per clinic in each of the 10 clinics (200 dogs in total). Dogs confirmed as new (previously undiagnosed) cases of OA by a veterinarian based on the owner‐completed checklist, clinical history and examination were considered eligible as long as they were dogs of any pure or mixed breed, of any size or sex, and older than 1 year of age, considered to have OA‐associated pain and appropriate candidates for at least 30 days of carprofen administration by the veterinarian, based on pre‐treatment laboratory work (i.e. complete blood count, chemistry panel, urinalysis) and physical examination. Confirmation of OA diagnosis was based on history, clinical examination and radiographs (as required), consistent with the typical approach in veterinary general practise settings (Belshaw *et al*. 2020). During screening of all dogs by physical examination, joint pain was also confirmed. Study exclusion criteria consisted of dogs less than 1 year of age, those that were already diagnosed with OA and receiving treatment, those that were receiving corticosteroid therapy for any reason, and/or those that were identified by the attending veterinarian as not being appropriate candidates based on pre‐screening laboratory work, physical examination and/or medical history.

### Risks and consent

In the protocol, any dog was able to be removed for any reason. No long‐term effect data were captured for dogs completing the study. Once a dog owner consented to participate in the study, the consulting veterinarian provided a complete blood count, chemistry panel and urinalysis to identify any pre‐existing conditions that would preclude the dog from being a candidate for carprofen administration. Instructions to the veterinarians were provided to report any suspected adverse events. Other data collected included clinic name, name of each patient, identification number, days of observation (0, 30 and 120 days) and location of limbs with OA.

### Treatment

Enrolled dogs were prescribed carprofen at the labelled target dose of 4.4 mg/kg (2.0 mg/lb) bodyweight, administered orally every 24 hours or divided into two daily doses administered every 12 hours, for at least 30 days. The drug dosage was not changed at any time during the study. Two of the 10 clinics agreed to continue treatment to 120 days.

### Functional test videos

**FIG 1:**
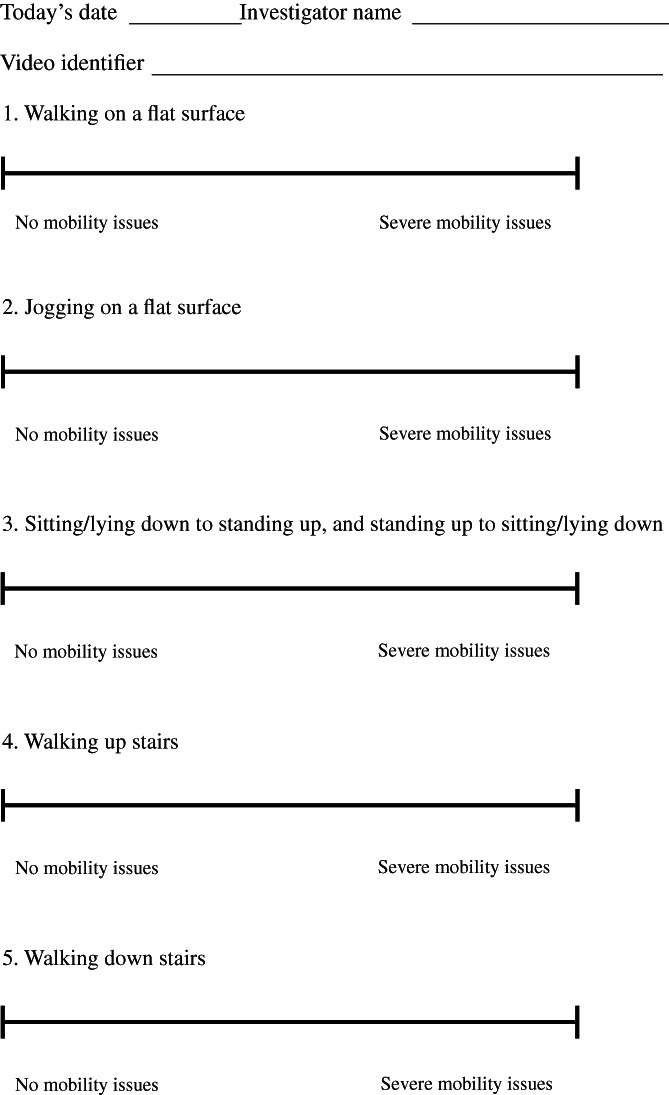
Visual analogue scale worksheet for scoring video recordings. Each visual analogue scale was 100 mm in length where zero was equal to no mobility issues and 100 was the most severe mobility score. Marks were measured and recorded for each dog for each of the five activities

On the day of OA diagnosis (before administration of carprofen), following 30 days (±5) of treatment (in the 30‐day group), and again following 120 days of treatment (in the 120‐day group), practise personnel collected videos of the dogs performing five activities of daily living: walking on a flat surface (front, side, rear views), jogging on a flat surface (front, side, rear views), rising from a sitting/lying position (side view), ascending stairs (front and rear views) and descending stairs (front and rear views). Written instructions for recording the videos (Table 2) were provided to the veterinary clinics. All clinic personnel were provided with an iPad mini 2 (16GB models with retina display) to capture digital videos. Personnel were instructed to limit the total length of each video (all views) to 2 minutes. Videos were uploaded to Vimeo® Basic App (Vimeo Inc., New York, USA) installed on the iPads. Videos were accessed on the Vimeo site, downloaded and renamed using randomly generated nine‐digit numbers. A key was created so unmasking of the scores could be performed. The videos were sent to the independent expert that was the blinded evaluator. Videos were scored by the evaluator using a VAS to quantify the dogs' ability to perform five activities, including walking, jogging, sitting/lying, walking up and down stairs (Fig 1). Each VAS was 100 mm in length where zero was equal to no mobility issues and 100 was the most severe mobility score. Marks were then measured and recorded for each dog for each of the five activities. These objective VAS ability scores were generated because lameness is very difficult to measure subjectively, especially when trying to assess a response to therapy by the veterinarian and the dog owner.

**Table 2:** Instructions for recording videos for ability analysis of daily activities in dogs diagnosed with OA before and after carprofen treatment

Activity	Video angle and instructions
Walking	Front shot: have the dog start walking toward you
	Side shot: continue filming as the dog walks past you
	Behind shot: finish the video with a rear view of the dog walking away from you
Jogging on a flat surface	The dog should take approximately 20 to 30 jogging steps in total
Sitting to rising/standing	The dog can start from either a sitting/lying down position
Walking up and down stairs	Front shot: have the dog start walking towards you down the stairs
	Behind shot: when the dog reaches the bottom, have him/her turn around and climb the stairs

OA Osteoarthritis

### Pain assessment by HCPI

Pain assessment was performed by the pet owner at baseline (day 0), 30 days and 120 days (if relevant) by completion of the HCPI questionnaire. Owners rank pain on a scale of 0 to 4 with greater severity of pain represented as a higher pain score, for a total of 11 different questions. The expert evaluator was blinded to the results of the HCPI questionnaire.

### Checklist evaluation

Data from the checklist questions (answered as yes or no) were analysed to determine their accuracy in identifying incident cases of OA in canine patients, in comparison to veterinary assessment. OA diagnosis performed by the veterinarian as standard was considered to refer to the true disease status. Sensitivity, specificity, accuracy, predictive positive value and negative predictive value and their 95% confidence intervals (CI) were computed for each question, as well as for answering yes to at least one of the nine questions of the checklist and to at least one of the six questions with sensitivity >40%, using the command *diagt* in Stata 12.0 (StataCorp LP, College Station, TX, USA).

### Ethical statement

The Zoetis Kalamazoo Ethical Review Board, a panel of objective experts, reviewed the proposed *in vivo* activities to ensure the use of animals was consistent with sound scientific practises, the 3Rs (Rychel 2010) and ethical considerations in compliance with local, regional and international regulations and guidance.

### Statistical analysis of VAS scores

Descriptive statistics [mean, median, standard deviation (SD) and range] for VAS ability scores were computed overall and by time of observation. Associations between time of observation (days 0, 30 and 120) and ability scores for walking and jogging were tested using linear mixed models fitted with a Gaussian distribution, identity link and residual pseudo‐likelihood in SAS (SAS 9.4, SAS Institute Inc., Cary, NC, USA). Time was modelled as an independent variable (categorical: 0, 30 and 120 days) and ability scores for walking and jogging were modelled as the dependent variables (in separate models). A random intercept for clinic was included, as well as a random residual component with a first‐order autoregressive heterogeneous covariance structure [also Toeplitz and unstructured covariance structures were tested and information criteria (Akaike's Information Criteria (AIC) and Bayesian Information Criteria (BIC)) were used to determine best model fit], to account for the design structure (dogs within clinics and repeated measures at the dog level) of the study. A similar procedure was followed to test associations between time of observation with outcomes pertaining to sitting and lying, walking up stairs and walking down stairs. Due to the lack of normality and homoscedasticity of these models' residuals, generalised linear mixed models were fitted using a Beta distribution, logit link, residual pseudo‐likelihood and Newton‐Rapson with Ridging optimisation algorithm. Dependent variables consisted of ability scores, which were transformed to continuous proportions from 0 to 1 (by dividing values by 100). Zeros were assigned a value of 0.005. The same random structure as described above was fitted in these models. Model assumptions were tested in all models and residuals were investigated at the different hierarchical levels using graphical tools. Mean ability scores and their 95% CI were computed. P values <0.05 were considered statistically significant. The Tukey–Kramer procedure was used to prevent inflation of type I error due to multiple comparisons. For this one‐arm, open‐label study, a sample size of 133 provides a power >80% to detect an increase of a mean ability score of at least five points, considering a significance level of 5%, SD of 20, one baseline measurement and two follow‐up measurements (and 70% correlation between measurements).

## RESULTS

### Owner‐reported OA screening checklist

#### Study population

Descriptive statistics of the study population and tabulation of demographic variables by OA diagnosis are presented in Table 3. A total of 500 owner‐reported checklists were completed. These yielded 188 (37.6%) previously undiagnosed OA cases confirmed by a veterinarian during the consultation.

**Table 3:** Descriptive statistics of the study population and tabulation of demographic variables by OA diagnosis

Variable, units	n	Mean	Median	Range	
Age, years^*^	490	7.67	7.6	1.0 to 20.0	
Weight, kg^†^	477	23.7	23.3	2.1 to 90.7	

Variable	n	%			
OA diagnosis					
OA	188	37.6			
Not OA	312	62.4			
Total	500	100.0			

				OA diagnosis	
				Yes	No
Therapy recommendation			Therapy	n (%)	n (%)
Yes	131	26.2	Yes	131 (75.3)	0 (0.0)
No	330	66.0	No	43 (24.7)	287 (100.0)
NA	39	7.8			
Total	500	100.0	Total	174 (100.0)	287 (100.0)

				OA diagnosis	
				Yes	No
Sex	n	%	Sex	n (%)	n (%)
FI	1	0.2	FI	1 (0.5)	0 (0.0)
FS	52	10.4	FS	52 (27.7)	0 (0.0)
MI	2	0.4	MI	2 (1.1)	0 (0.0)
MN	46	9.2	MN	45 (23.9)	1 (0.3)
NA	399	79.8	NA	88 (46.8)	311 (99.7)
Total	500	100.0	Total	188 (100.0)	312 (100.0)

				OA diagnosis	
				Yes	No
Breed group^‡^			Breed	n (%)	n (%)
Gundog	132	26.4	Gundog	56 (29.8)	76 (24.4)
Hound	40	8.0	Hound	17 (9.0)	23 (7.4)
Pastoral	58	11.6	Pastoral	18 (9.6)	40 (12.8)
Terrier	47	9.4	Terrier	21 (11.2)	26 (8.3)
Toy	59	11.8	Toy	18 (9.6)	41 (13.1)
Utility	63	12.6	Utility	30 (16.0)	33 (10.6)
Working	55	11.0	Working	14 (7.5)	41 (13.1)
Other	42	8.4	Other	14 (7.5)	28 (9.0)
NA	4	0.8	NA	0 (0)	4 (1.3)
Total	500	100.0	Total	188 (100)	312 (100)

OA Osteoarthritis, NA Not available/not answered/missing

^*^ Age for 10 dogs was not recorded

^†^ Weight for 23 dogs was missing

^‡^ Breeds were categorised according to The Kennel Club breed groups (https://www.thekennelclub.org.uk/breed‐standards/). When depicted as mixed breed, a category was assigned based on the first breed specified. Breeds defined as Other corresponded to breeds that were not presented within The Kennel Club list of breeds

#### Checklist evaluation

Table 4 depicts contingency tables between each of the checklist questions and OA diagnosis, as well as sensitivity, specificity, accuracy, positive predictive value and negative predictive value and their corresponding 95% CI. Sensitivity and specificity for the nine questions in the checklist as a group, answering yes to at least one of the questions, were approximately 99 and 36%, respectively. After elimination of three questions with the lowest sensitivity (≤40%) (Q7 to Q9), sensitivity and specificity for the remaining questions in the checklist as a group, answering yes to at least one of the questions, were approximately 88 and 71%, respectively. These three questions with the lowest sensitivity referred to historic events or circumstances that may contribute to OA risk rather than being indicative of current OA‐related behaviours.

**Table 4:** Sensitivity, specificity, accuracy, positive and negative predictive values for each checklist question, across six of the questions and across the original nine questions

Question	OA diagnosis			Se (95% CI) (%)	Sp (95% CI) (%)	Accuracy (95% CI) (%)	PPV (95% CI) (%)	NPV (95% CI) (%)
	Yes	No	Total					
Q1. Limp or stiff after exercise				63.4 (56.0 to 70.4)	88.8 (84.7 to 92.1)	79.4 (75.6 to 82.9)	76.8 (69.3 to 83.3)	80.5 (75.9 to 84.6)
Yes	116	35	151					
No	67	277	344					
Total	183	312	495					
Q2. Signs of pain				64.3 (56.9 to 71.2)	86.5 (82.1 to 90.1)	78.3 (74.3 to 81.8)	73.6 (66.0 to 80.3)	80.5 (75.8 to 84.6)
Yes	117	42	159					
No	65	268	333					
Total	182	310	492					
Q3. Reluctant to climb stairs or jump				68.1 (60.9 to 74.8)	84.6 (80.1 to 88.4)	78.4 (74.5 to 82.0)	72.4 (65.1 to 78.9)	81.7 (77.0 to 85.7)
Yes	126	48	174					
No	59	263	322					
Total	185	311	496					
Q4. Difficulty in rising from resting position				57.3 (49.8 to 64.5)	86.7 (82.4 to 90.3)	75.7 (71.6 to 79.4)	72.1 (64.1 to 79.2)	77.2 (72.4 to 81.5)
Yes	106	41	147					
No	79	267	346					
Total	185	308	493					
Q5. Change in dog behaviour				40.9 (33.6 to 48.4)	90.0 (86.1 to 93.1)	71.8 (67.6 to 75.8)	70.5 (60.8 to 79.0)	72.2 (67.4 to 76.6)
Yes	74	31	105					
No	107	278	385					
Total	181	309	490					
Q6. Lag behind during walks				54.9 (47.4 to 62.2)	85.8 (81.4 to 89.5)	74.3 (70.2 to 78.1)	69.7 (61.5 to 77.0)	76.2 (71.4 to 80.6)
Yes	101	44	145					
No	83	266	349					
Total	184	310	494					
Q7. Injured before				35.9 (28.9 to 43.3)	83.3 (78.7 to 87.3)	65.7 (61.4 to 69.9)	55.9 (46.5 to 65.1)	68.8 (63.8 to 73.4)
Yes	66	52	118					
No	118	260	378					
Total	184	312	496					
Q8. Medication for pain				39.1 (32.0 to 46.6)	81.0 (76.1 to 85.2)	65.4 (61.0 to 69.6)	55.0 (46.0 to 63.7)	69.1 (64.1 to 73.9)
Yes	72	59	131					
No	112	251	363					
Total	184	310	494					
Q9. Gained weight				40.0 (32.9 to 47.4)	62.7 (57.0 to 68.1)	54.2 (49.6 to 58.6)	39.2 (32.2 to 46.5)	63.5 (57.8 to 68.9)
Yes	74	115	189					
No	111	193	304					
Total	185	308	493					
At least one of the first six questions (Q1 to Q6) was answered with a “yes”				88.2 (82.6 to 92.4)	70.5 (65.1 to 75.5)	77.1 (73.2 to 80.7)	64.1 (57.9 to 69.9)	90.9 (86.6 to 94.2)
Yes	164	92	256					
No	22	220	242					
Total	186	312	498					
At least one of the nine questions (Q1 to Q9) were answered with a “yes”				98.9 (96.2 to 99.9)	36.5 (31.2 to 42.1)	59.8 (55.4 to 64.2)	48.2 (43.1 to 53.3)	98.3 (93.9 to 99.8)
Yes	184	198	382					
No	2	114	116					
Total	186	312	498					

OA Osteoarthritis, Se Sensitivity, CI Confidence intervals, Sp Specificity, PPV Positive predictive values, NPV Negative predictive values

### Functional tests study

#### Descriptive statistics

A total of 399 video scores were collected from 133 dogs with a range of three to 36 cases per clinic. Data on the location of OA were available for 68% (90 of 133) of dogs, with the majority (59%, 53 of 90) affected bilaterally in the hind limbs (Table 5). The mean, median, SD and range of ability scores by time of observation are presented in Table 6.

**Table 5:** Location of OA

Location of OA	% (n)
All limbs and back/spine	1 (1/90)
Bilateral hind limbs and back/spine	1 (1/90)
Bilateral front and hind limbs	11 (10/90)
Front limb and bilateral hind limbs	4 (4/90)
Bilateral hind limbs	59 (53/90)
Single hind limb	14 (13/90)
Single front limb	6 (5/90)
Back/spine only	3 (3/90)

OA Osteoarthritis

Data available for 90 of 133 dogs. The joints identified with OA pain in the hind limbs were hip, stifle, tarsus and metarsophalangeal joint. In the front limbs, the joints evaluated for OA pain included shoulder, elbow and carpus

**Table 6:** Descriptive statistics for ability scores^*^ of daily activities in dogs diagnosed with OA before and after carprofen treatment

Activity	Time (days)	n	Mean	Median	SD	Range
Walking on a flat surface	0	126	26.4	27.0	17.7	0 to 72
	30	122	22.2	21.5	16.2	0 to 60
	120	38	23.6	21.5	15.4	0 to 57
Jogging on a flat surface	0	127	26.8	28.0	19.3	0 to 100
	30	120	21.7	20.0	16.8	0 to 74
	120	38	24.2	21.0	19.5	0 to 100
Sitting/lying down	0	115	18.0	17.0	18.4	0 to 63
	30	102	13.8	6.5	16.7	0 to 74
	120	31	11.6	0	16.2	0 to 48
Walking up stairs	0	90	26.6	23.5	24.1	0 to 100
	30	84	22.9	20.0	23.3	0 to 100
	120	15	16.9	16.0	19.8	0 to 73
Walking down stairs	0	88	20.6	16.0	22.4	0 to 100
	30	82	17.6	13.5	20.2	0 to 100
	120	14	19.9	17.0	15.6	0 to 51

OA Osteoarthritis, n Number of observations, SD Standard deviation

^*^ Ability scores, measured using a visual analogue scale method, were recorded on a scale from 0 (no ability issues) to 100 (severe ability issues)

#### Videotape mobility assessment

Data for the videotaped mobility assessment determined by videotape analysis of mobility using VAS scores are shown in Tables 7 and 8. At baseline, most dogs displayed impaired mobility for each behaviour, which ranged from 59% having difficulty rising from sitting/lying, and 85 and 86% experiencing difficulty jogging or walking, respectively (Table 7). At day 30, improved impairment percentages were observed for each mobility category, particularly a significant decrease by 26% of dogs affected when jogging or climbing stairs. Improvements were observed in each mobility category, except climbing stairs, by day 120 from baseline (Table 7). Duration of treatment had a significantly positive effect on all five functional tests. Mean ability scores and their 95% CI, differences in means and P values by time of observation are depicted in Table 8. Briefly, all ability scores were highest (poorest ability) on day 0. Scores were lowest on day 30 for walking, jogging and walking down stairs. Scores were lowest on day 120 for sitting/lying and walking up stairs. Mean ability scores between days 0 and 30 were significantly different for walking, jogging, sitting/lying and walking up stairs (Table 8). Mean ability between days 0 and 120 were significantly different for sitting/lying and walking up stairs (Table 8). Mean ability between days 30 and 120 was significantly different for walking up stairs (Table 8).

**Table 7:** Mobility assessment of osteoarthritic dogs treated with carprofen as determined by visual analogue scale scoring of video recordings

	Day 0		Day 30		Day 120	
Mobility behaviour	Proportion (%)	95% CI (%)	Proportion (%)	95% CI (%)	Proportion (%)	95% CI (%)
Walking	108/126 (85.7)	78.4 to 91.3	66/119 (55.4)^*^	46.1 to 64.6	16/36 (44.4)	27.9 to 61.9
Jogging	108/127 (85.0)	77.6 to 90.7	70/118 (59.3)^†^	49.9 to 68.3	15/35 (42.9)	26.3 to 60.6
Sitting/lying down	67/114 (58.8)	49.2 to 67.9	47/95 (49.5)^‡^	39.1 to 59.9	8/22 (36.4)	17.2 to 59.3
Climbing stairs	66/90 (73.3)	63.0 to 82.1	33/78 (42.3)^§^	31.2 to 54.0	9/15 (60.0)	32.3 to 83.7
Going down stairs	58/89 (65.2)	54.3 to 75.0	37/76 (48.7)^‡^	37.0 to 60.4	6/13 (46.2)	19.2 to 74.9

CI Confidence interval

Based on visual analogue scale 0 to 100 scores

^*^ P=0.09 *versus* day 0

^†^ P=0.01 *versus* day 0

^‡^ P>0.70

^§^ P=0.05 *versus* day 0

**Table 8:** Model‐adjusted mean ability scores, differences in means, 95% CIs and P values by time of observation

Activity	Time (days)	Mean ability score	95% CI mean ability scores	P value^*^	Contrasts	Mean differences in mean ability scores	P value^†^
Walking on a flat surface	0	26.46	22.81 to 30.10	0.005	0 *versus* 30	4.43	0.004
	30	22.03	18.56 to 25.50		0 *versus* 120	4.05	0.17
	120	22.41	17.48 to 27.33		30 *versus* 120	−0.38	0.98
Jogging on a flat surface	0	26.57	22.36 to 30.79	0.006	0 *versus* 30	4.97	0.004
	30	21.60	17.69 to 25.52		0 *versus* 120	2.94	0.58
	120	23.63	17.27 to 30.00		30 *versus* 120	−2.03	0.75
Sitting/lying	0	17.74	14.82 to 21.08	<0.001	0 *versus* 30	5.11	<0.001
	30	12.63	10.26 to 15.46		0 *versus* 120	6.78	<0.001
	120	10.96	8.14 to 14.62		30 *versus* 120	1.67	0.49
Walking up stairs	0	24.50	21.28 to 28.03	0.003	0 *versus* 30	2.50	0.25
	30	22.00	18.90 to 25.45		0 *versus* 120	10.22	0.003
	120	14.28	9.94 to 20.08		30 *versus* 120	7.72	0.025
Walking down stairs	0	18.86	15.98 to 22.13	0.03	0 *versus* 30	2.95	0.023
	30	15.91	13.24 to 19.00		0 *versus* 120	1.20	0.86
	120	17.66	13.26 to 23.14		30 *versus* 120	−1.75	0.69

CI Confidence intervals

^*^ Overall significance test (*F* test)

^†^ P values represent Tukey–Kramer's adjustment for multiple comparisons. P values <0.05 represent statistically significant differences in ability scores between times

#### HCPI pain measurements

Across study dogs, the client‐generated, mean total HCPI score at baseline was 18.74, with an average individual item score of 1.7 (Table 8). The range in mean values was one to 34. The wide range in mean values (1 to 34) at baseline reflects a general presence of pain‐associated behaviours in the study population. Some animals exhibited severe pain‐related signs of OA, shown by a baseline total HCPI score of 34. A decrease in the average HCPI score by 6.22 points (33.2%) to 12.52 was demonstrated by day 30, with a comparable decrease in the average individual item score to 1.1. In comparison to HCPI definitions, an individual item score of 1.1 indicates that carprofen‐treated dogs had a high level of pain‐free functionality. A marginal rise in HCPI by 0.7 points was observed from day 30 to 120. The 5.52‐point (29.5%) improvement in the mean HCPI score from day 0 to 120 indicated that dogs treated with carprofen for the duration of the study had a sustained therapeutic response.

## DISCUSSION

Key findings from this study revealed that client responses to the OA completed checklist assisted in identifying 188 (38%) previously undiagnosed cases of OA, confirmed by physical or radiographic examination, from 500 previously undiagnosed dogs attending non‐acute healthcare visits, or specifically presented because of stiffness or lameness. The proposed checklist represented a starting point for discussion with owners and further veterinary investigation. We report prevalence (38%) at almost double that of previous, widely cited prevalence estimates of 20% for OA in the canine population (Johnston 1997). In addition, less than half (n=60, 47.2%) of the enrolled dogs presented for stiffness or lameness, irrespective of the fact that all enrolled dogs were previously undiagnosed cases of OA. This indicates that OA is considerably underdiagnosed in dogs, and far more prevalent in the canine population than previous estimates suggest, likely due to signs going unrecognised by owners and veterinarians.

Screening tests are not considered diagnostic but are used to identify a subset of the population that should undergo further investigation in order to accurately establish the presence or absence of disease. Use of a simple, well‐targeted questionnaire, based on simple questions of canine behaviours indicative of mobility impairment, proved effective in identifying for further evaluation cases OA that could otherwise remain undiagnosed. The sensitivity and specificity of the nine‐question screening checklist were approximately 99 and 37%, respectively. After elimination of three questions, sensitivity and specificity were approximately 88 and 71%, respectively. Identification of previously undiagnosed OA cases allows veterinarians to initiate a treatment intervention to manage pain and improve quality of life. To our knowledge, owners completing the checklist in the present study had not been previously educated about OA in dogs. A previous study using a DJD screening checklist in cats reported that accuracy of the checklist was substantially higher when completed by informed owners who were aware of DJD and associated pain (Enomoto *et al*. 2020). This represents an opportunity for engagement and education of owners with adult and senior dogs. As dogs are most likely to perform behaviours at home rather than at the clinic, engagement is critical to the detection and diagnosis of OA and associated pain.

Functional tests do not appear to have been previously described before in veterinary medicine for dogs with OA. These five functional tests of common daily activities of walking, jogging, sitting/lying and walking up and down stairs were efficient to perform in a veterinary clinic and record *via* video on an iPad and store for evaluation. These tests were not validated so the assessment was blinded. Future work should verify the findings using placebo controls and report methods to objectively measure the difference in response to treatment.

Although there was no placebo group in this study due to the ethical concerns with diagnosing OA and not providing pain relief for 30 days or up to 120 days, bias resulting from knowledge of the treatment for both the veterinarians and owners was countered by the complete de‐identification of videos and blinding of the independent evaluator for the generation of VAS scores when assessing the outcomes. The videos were analysed by assessing the dogs' ability to perform the activities, smoothness, ease and overall assessment of the entire animal.

Data showed that carprofen improved dogs' ability to perform five activities of daily living when administered over a 30‐day period (30‐day group; n=95), and continued treatment up to 120 days (120‐day group, n=38) resulted in additional improvement in certain activities. The activities that improved with continued treatment up to 120 days were rising from sitting/lying and walking up stairs. Our assumption is that these are more painful than walking and trotting in a straight line or walking down stairs. These activities measured are relevant as they are encountered by most dogs in their daily home environment. The most difficult activities of going up stairs and rising from sitting/lying appear to take longer to improve. In addition, location of limb data was available for 90 dogs and only five dogs had OA diagnosed in one front limb only. Going up stairs and rising from sitting/lying involves more strength and weight on joints in the hindlimbs and in 60% of the patients OA was identified in the bilateral hindlimbs. Most dogs where location of the limb data was available included hind limbs and or back/spine which would make going up stairs and rising from sitting/lying more difficult. The high prevalence of bilateral hindlimb OA in this study population would also make recognition of OA in these cases more difficult than cases where only one limb is involved showing more obvious clinical signs of lameness.

A limitation of this study consisted of the lack of a complete set of videos from all veterinary clinics, which left evaluators unable to score a full set of activities for all dogs. Forty percent of videos were not scored due to the absence of some views required for scoring, but all dogs had at least three activities available for VAS scoring. In one clinic, no stair activities were videotaped, contributing largely to the missing scores and fewer dogs analysed in these activities. There were also fewer dogs in each activity recorded at day 120 as only two veterinary clinics chose to participate for 120 days and more dogs were lost to follow‐up in these clinics. If more dogs had videos collected at 120 days, a more complete picture of the degree of this drug's effect on ability over this longer time span may have been seen. Additional studies could be improved by using a more advanced model by including the nested effect of dogs to the clinics. In other words, the structure of dogs nested in the clinic should be considered. To improve the quality of the videos, it is now widely recommended to take the videos in slow motion. The surfaces that the videos were recorded differed in hardness that may have impacted the mobility issues seen but the surface hardness was consistent for each individual dog in each individual video. Another limitation is that radiographic confirmation was not available for all dogs in this study. The location of the limbs involved was confirmed for 90 dogs but not for all dogs. We cannot be certain that all dogs had decreased ability to perform activities due to OA only. Diagnosis was based on history, clinical examination, and radiographs (as required), as typically performed in veterinary general practise settings (Belshaw *et al*. 2020). All dogs were screened by physical examination and appeared to have joint pain. In future studies, diagnosis of OA with an orthopaedic examination, joint pain and confirmatory radiographic evidence would improve the study design. Additionally, the label for carprofen allows for dosing either once or twice a day. To our knowledge, there are no scientific data that suggest that the clinical outcome would be different between once and twice a day treatment. To improve future studies, researchers could investigate differences in the clinical outcomes for functional tests described with once or twice a day dosing. A further study limitation is that it is possible the participating veterinary clinics' interest in participating in the study may have created a self‐selection bias toward a higher reported prevalence of OA.

We conclude that prevalence estimates of canine OA are likely much higher than previously reported. This should be confirmed with comprehensive radiographic and clinical evaluation studies in a large cohort of dogs. Use of an owner‐completed checklist was confirmed as an effective initial screening tool for identifying OA‐related behaviours, indicative of the presence of previously undiagnosed OA in canine patients. When coupled with educational tools designed to engage owners in monitoring their dogs for behaviours associated with OA pain, the checklist has potential to provide a foundation for increasing awareness of OA among dog owners and increase veterinarian's ability to screen for OA in a clinically expedient manner. Early detection and treatment intervention for OA has the potential for veterinarians to enhance the wellbeing of their canine patients. Video mobility analysis indicated that ability to perform functional tests of daily living (walking, jogging, sitting/lying, walking down stairs and walking up stairs) significantly improved over a 30‐day period in dogs newly diagnosed with OA and treated with carprofen. For the two activities of sitting/lying and walking up stairs, a positive treatment response was maintained between 30 and 120 days, and this improvement was significant for climbing stairs. These results highlight the benefits of long‐term treatment and regular monitoring in a clinical setting for dogs with OA‐associated impairment of ability in performing activities of daily living. Video recording these functional activities of daily living combined with pain assessment tools and re‐evaluating after therapy may assist with ongoing treatment decisions and discussions with dog owners about response to therapy.

### Funding

This study was funded by Zoetis.

### Conflict of interest

AW, DB and DMA are full‐time employees of Zoetis. NC was remunerated for the advanced statistical analysis. BDXL was remunerated for the time taken to score the videos. AP provided independent contracting services for Zoetis.

## Data Availability

The datasets are available upon request to the corresponding author.
